# Review of Current Neurological Matters

**Published:** 1877-08-20

**Authors:** C. C. Vanderbeck


### REVIEW OF CURRENT NEUROLOGICAL MATTERS.

CONDENSED BY C. C. VANDERBECK, M.D.,

In *American Journal* of the *Medical Sciences* for January 1877, Horatio C. Wood, M.D., has detailed a case of "Partial Aphasia without appreciable lesion of Island of Reil."

Post mortem examination revealed no change in the Island of Reil, though the membranes over it were thickened. Some other pathological conditions were found, such as thickening of Dura Mater, great increase of Cerebro-Spinal Fluid, thickening and exudation of lymph, behind the optic chiasm. Softening of extreme anterior apex of middle lobe on right side. During life ophthalmascopic examinations revealed no choked disk. Dr. Wood takes occasion to remark that he doubts the supreme diagnostic value of choked disks. Its absence does not prove the "non existence of cerebral growths, or even of meningitis." He suggests that this case "does not disprove the theory of speech localization. The Island of Reil secures much of its blood-supply from the superjacent meninges," and as these were thickened, it is possible that sufficient disturbance was produced in the circulation of the part as to cause interference of function.

### PREVALENCE AND FATALITY OF NERVOUS DISEASES.

Dr. Julius Althaus, of London, has drawn up a series of diagrams and tables, based upon extensive statistics, illustrating these questions. The result may be closely condensed thus: Paralysis has more largely and steadily increased than either cephalitis or apoplexy. Delirium tremens seems, on the whole, to be declining at the present time. Cephalitis, including the various inflammatory diseases of the brain and spinal cord, and their membrane, excepting tubercular meningitis, show an increase in the fatality of late years.

The popular notion that the mortality from *insanity* has increased during the last ten years, is shown to be correct. Convulsions, chiefly infantile eclampsia, form by far the most important cause of death amongst the several diseases of the nervous system. Diseases of the brain, including a variety of nervous maladies, show a tolerably steady rise, corresponding with the increase to be expected by reason of increase of population. The several diseases of the nervous system arranged according to their fatality are: Convulsions, apoplexy, paralysis, disease of brain, cephalitis, epilepsy, insanity, delirium tremens, tetanus, chorea. In answer to the question, "are nervous diseases, as is commonly asserted, more frequent in large towns than in the country, and is there any apparent influence of race or climate," he is of the opinion that the general notion is fallacious. He collected and compared the deaths from these diseases as they occurred respectively in London, the South-western counties, and Wales. He finds the death rate *lowest* in London; highest in Wales. He concludes "that excess of *manual* labor is more exhaustive to the nervous system than excess of mental labor." The Welsh are the highest, not only by reason of habitation and occupation, but by reason of difference of race. The nervous system of the Anglo-Saxon has greater powers of endurance and resistance to unfavorable influences than that of the Celtic races. This may furnish a clue why the Anglo-Saxon race appears destined to rule the world. This same gentleman discusses the *influence of age on the fatality of nervous diseases.* He shows that cephalitis is more common in infancy than later in life—the first year being the most fatal. Apoplexy is not uncommon in the first year, but declines afterwards and reaches a minimum at ten to fifteen; begins to rise at thirty-five, and a maximum attained at seventy. Paralysis carries off very few in early life; the maximum is reached at seventy. Delirium tremens has no deaths before fifteen; the maximum occurs at thirty-five. Chorea appears to be most fatal between five and fifteen years of life. Tetanus is comparatively frequent in the first year. A slight fall in the second year, in the the third a slight rise, and the mortality of the period under five years of age, is altogether the greatest. Epilepsy to be very fatal in the first year, and altogether so in the first lustrum of life. Insanity carries off a few cases at two years of age; sixty-five years of age being the maximum.

In convulsions the influence of age is, perhaps, more apparent than in any other disease. The diagramatic representation shows that the curve commences almost at the top in the first year of life; falls with great rapidity in the second, third, fourth and fifth year, and reaches the summit in the first lustrum of life. After that it descends with unparalleled precipitation, never to rise again. Convulsions during the rest of life are of only very slight account in the mortality from nervous diseases.

SUPCUTANEOUS INJECTION OF MORPHIA FOR SCIATICA, LUMBAGO, AND BROCHIALGIA.—Dr. Henry Lawson, of London, writing in the London Times & Gazette, expresses his confidence in this mode of treatment as to relief of pain, but is not so positive of its radical curative worth. During the patient s freedom from pain, he pays every attention "to his feeding, his walking, his warmth, and his *ease of mind.*" No bodily improvement can go on with a worried state of mind. For a constitutional treatment he uses cod-liver oil, perchloride of iron, and hypophosphite of soda. He thinks this last salt one of the best ways to introduce phosphorus into the system. Some hints of value are given as to the use of the hypodermic injection:

1st. It is important to make the injection as close to the seat of pain as possible.

2nd. The needle of the instrument should be short. The long needle is more apt to produce abscesses, and absorption is not so rapid when this is used, "for the portion of the integument immediately beneath the 'zone of indifferent tissue,' is loaded with minute bloodvessels, while further in you have merely loose connective tissue, with far less vascularity. Again, the short needle is not so apt to break.

3d. Steel needles are preferable to gold; they are less easily broken, and keep their point better, and they are driven with less force.

4th. Never forget to clean the instrument after use. The doctor then investigates the question, Does bleeding from the wound inflicted by the syringe tend to render the absorption of the morphia more rapid than it would otherwise be? He believes that when a minute vein "has been opened in the course of the injection, a much more rapid absorption takes place."

5th. It is advisable that the patient should have eaten a good meal at least half an hour before the injection, as thus the soporific effect ot the drug is much decreased.

In two or three cases the eyes have become curiously affected after the injection; one pupil more contracted than the opposite one, and marked impairment of vision in the contracted eye. The patient, in each case a male, can not see either near or distant objects distinctly, when looking from this one eye. This condition may last a number of hours.

· FIRST DENTITION.—Dr. Little, of London, regards the evils of dentition "as the product of unfavorable influences in operation either before or at birth, or about the teething period." Infants who lack sufficient breastmilk, pure air, etc, "become the subjects of difficult dentition, rickets, infantile paralysis, breast and brain disease." "He thinks that the accumulated hindrances to development, occuring during the early months of life, produce effects during dentition which are often attributed to it instead of to disorder of more important organs, consequent upon errors of management or hereditary influence." He, at the same time, does not doubt the evil influence of difficult dentition upon the hyper-sensitive nervous structure of the infant.

West believes, that to dentition, directly, might be ascribed many diseases, especially those connected with the nervous centres. He mentions the great predominance of the spinal over the cerebral system in early infancy, and shows how any eccentric source of irritation was thus liable to be the origin of serious mischief.

SYMPATHETIC LESION CONNECTED WITH THE TEETH.

Dr. Hamilton Cartwright had only recently seen a case where the extraction of a tooth had relieved supposed amaurosis. He has under his care a lady who invariable referred pain to certain teeth on the recurrence of each menstrual period. Otalgia and Otorrhoea during dentition can be explained by the connexion of the auricula—temporal and vidian nerve with the fifth, giving pain in the ear, and the long continued irritation of Jacobson's flexus, which, supplying the tympanic structures, is in connection with the otic and mechele ganglia, as also with the facial nerve and the carotid branch of the sympathetic. This explains suppuration of the ear.

DEATH OF A CHILD FROM FRIGHT.—The London *Medical Times and Gazette* notes such a case: The girl was but four years of age, and was so frightened by being shut up in a dark cupboard at a school, that "paralysis of the throat was caused, and death ensued.

[*To be Continued.*]

